# Case Report: Mesh repair of a urinary bladder dorsal wall defect following repeated cystorrhaphy in a warmblood foal

**DOI:** 10.3389/fvets.2026.1716790

**Published:** 2026-03-31

**Authors:** Rieke S. Gehrke, Anna Ehrle, Eva M. T. Müller, Ronja-Katharina Kraul, Philip Schmitz, Christoph J. Lischer

**Affiliations:** Equine Clinic, School of Veterinary Medicine, Freie Universität Berlin, Berlin, Germany

**Keywords:** case report, cystorrhaphy, foal, mesh repair, urography, uroperitoneum

## Abstract

A three-day-old male Warmblood foal presented with inability to urinate. Primary diagnostic findings included abdominal distension, hyperkalemia, azotemia, and ultrasonographic evidence for free anechoic fluid in the abdominal cavity. Abdominocentesis confirmed the diagnosis of an uroperitoneum [peritoneal fluid to serum creatinine ratio (>2:1)]. Suture repair of a dorsal urinary bladder wall defect was performed, but leakage along the suture tracts resulted in recurrent uroperitoneum confirmed by computed tomographic urography after two days. Repeated cystorrhaphy was performed but uroperitoneum recurred four days later. The vesical tissue at the level of the tear appeared markedly inflamed and friable during the subsequent revision. The bladder wall defect was therefore reinforced with a monofilament (Monocryl-Prolene) composite mesh to prevent excessive bladder distension. The foal was discharged from the hospital three weeks after admission in good general condition. Telephone follow-up confirmed physiological development, urination and weight-gain of the animal. The horse deceased peracutely one year after discharge where urolith formation had caused urine leakage at the ventral aspect of the urinary bladder. The mesh was found to be integrated in the intact dorsal bladder wall without evidence for adhesion formation. This case report presents an alternative approach for managing the complication of urinary leakage associated with cystorrhaphy. Mesh repair should be considered early in cases where the vesical tissue is friable and primary suture repair at high risk of failure.

## Introduction

Uroperitoneum is a common and life-threatening condition in neonatal foals presenting at referral level (1, 2). The accumulation of urine in the abdomen is usually associated with a defect in the urinary bladder (1, 3, 4), with dorsal wall defects observed more frequently when compared to ventral wall defects (3). Based on the available literature, it remains unclear whether uroperitoneum occurs more frequently in male foals (2, 4, 5) or whether the condition is gender independent (1). The etiology has been described to be of traumatic, septic or congenital origin (6).

Foals with uroperitoneum regularly present with hyperkalemia, hyponatremia, hypochloridemia (1, 4) and azotemia (5–7), due to the resulting osmotic shift. The final diagnosis is based on the ultrasonographic examination of the abdomen (1, 8) as well as the serum creatinine level and the peritoneal fluid to serum creatinine ratio (>2:1) (1, 5). Ultrasonography is mainly used to visualize the anechoic free fluid within the abdominal cavity rather than the defect itself (1, 8). A retrograde contrast cystography can additionally be performed to confirm the diagnosis (9).

Prompt and consistent therapy is required for foals with urinary bladder rupture (1). Following stabilization of the patient including intra-venous fluid therapy to maintain ionic balance and drainage of urine from the peritoneal space as required, surgical cystorrhaphy is the treatment of choice (10). Recurrence of a bladder wall defect is the most common postoperative complication (3, 11). Uroperitoneal recurrence may be associated with leakage along the sutures in the bladder wall or suture dehiscence (1, 11). A postoperative recurrence rate of 12–20% has been described following surgical cystorrhaphy in foals (1, 3, 4).

Conservative therapy may be attempted in small lesions (1) or critically ill foals in order to avoid general anesthesia (9). Drainage of the abdominal cavity can be performed to evacuate the accumulated urine (12). The bladder should further be catheterized to reduce distension and urine outflow (9, 12). However, adhesion formation is a potential long-term complication after conservative and surgical treatment (12).

In equine surgery, prosthetic meshes are utilized for repair of abdominal wall defects (13, 14), diaphragmatic hernias (15), patella luxation (16) or to close the nephrosplenic space (17, 18). In a dog, a perineal hernia with a rectal diverticulum was repaired with a polypropylene mesh (19). In human surgery the monocryl-prolene mesh (Ultrapro™, Ethicon) has been used for hernia repair (20, 21) and to support direct breast reconstruction in breast cancer patients (22). The lightweight, partially resorbable mesh has good biocompatibility with a low local inflammatory response (23).

The aim of this case report was to document an alternative method for revision surgery in recurrent urinary leakage following repair of urinary bladder defects in foals. To the best of the authors’ knowledge the use of a prosthetic monocry-prolene mesh for cystorrhaphy has not been described in equine patients so far.

## Case description

### Examination and diagnosis

A three-day-old male Warmblood foal was referred as an emergency due to lack of urination. Parturition was reportedly free of complications, and the foal was observed to void urine in small quantities until the day of referral, when urination was not observed. Urinary bladder rupture was suspected.

On initial examination the foal was quiet but responsive. The umbilicus was dry but marked abdominal distention was evident. The oral mucous membranes were pale pink with a capillary refill time of <2 s (24). Physical examination further identified tachycardia (120 beats/min) and tachypnea (92 breaths/min). Hematology and blood biochemistry showed hemoconcentration (packed cell volume 42%; total protein 7.8 g/dL), azotemia (urea 74.7 mg/dL; creatinine 3.42 mg/dL) and hyperkalemia (potassium 5.4 mmoL/L). The serum IgG concentration was 7.9 g/L suggesting adequate colostrum intake (25). At this stage differential diagnoses included urinary tract abnormalities like lesions of the urinary bladder or ureters. Other possible but less likely differential diagnoses were gastrointestinal disorders such as meconium impaction, ileus or atresia coli, as well as systemic conditions including neonatal sepsis.

Ultrasonographic examination showed free anechoic fluid within the abdominal cavity and abdominocentesis confirmed the diagnosis of an uroperitoneum (peritoneal fluid urea 51.8 mg/dL; creatinine >10 mg/dL). Urinary drainage of the abdominal cavity was performed, and the foal was stabilized with intravenous fluid therapy (1 liter 0.9% sodium chloride with 50 mL 40% glucose substitution) prior to surgical repair of a suspected urinary bladder tear. Flunixin meglumine (1.1 mg/kg BW IV) and broad-spectrum antimicrobials (amoxicillin 20 mg/kg BW IV and amikacin 25 mg/kg BW IV) were administered.

### Initial surgery

Surgical cystorraphy was performed to directly address the suspected urinary bladder tear and ensure further clinical stabilization of the foal’s ionic imbalance. For premedication, butorphanol (0.05 mg/kg BW IV) and diazepam (0.05 mg/kg BW IV) were administered. General anesthesia was induced with ketamine (2.5 mg/kg BW IV). The foal was intubated with the aid of a laryngoscope, and anesthesia was maintained with isoflurane in oxygen (minimum alveolar concentration 0.7%). Following induction, the animal was positioned in dorsal recumbency, a urinary catheter was placed, and the prepuce and penis were subsequently reflected caudally to facilitate surgical access to the caudoventral abdomen. The surgical site was aseptically prepared with an iodine scrub (75 mg Povidon iod) followed by surgical spirit (Ethanol 70%). Routine sterile draping was applied with only the ventral midline remaining exposed.

**Figure 1:**
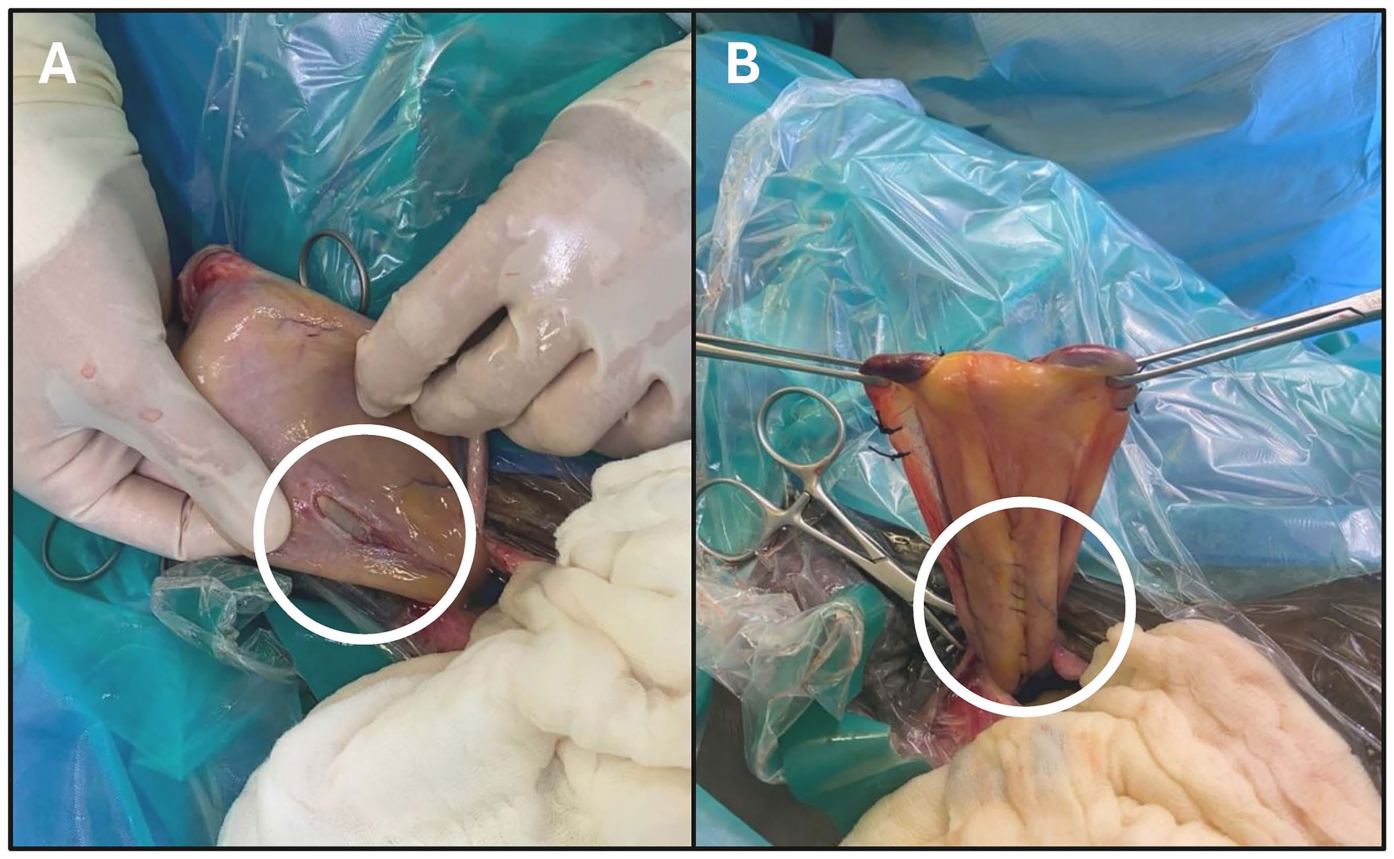
Intra-operative photographs obtained during the initial surgery prior to **(A)** and during **(B)** surgical repair on an approximately 4 cm dorsal vesical wall tear (white circle).

Following aseptic preparation, surgical cystorrhaphy was performed as previously described (26). In brief ventral midline celiotomy was performed, starting with a fusiform incision around the external umbilicus for removal. The subcutaneous incision was extended caudally, abaxial to the penis and prepuce. The prepuce was mobilized and retracted caudolaterally for deep access and incision of the linea alba in midline. After entering the abdominal cavity peritoneal fluid and urine were evacuated slowly using pool suction. The umbilical vein and the umbilical arteries were double-ligated (0 USP polyglactin 910) and transected. Inspection of the urachus and bladder identified an approximately 4 cm tear in the dorsal bladder wall (Figure 1A). Following sharp debridement of the wound margins, repair of the tear was performed with a double-layer of continuous inverting Cushing suture patterns (3-0 USP atraumatic poliglecaprone 25) (Figure 1B) avoiding the vesical mucosa. The umbilicus and urachus were removed and the cranial pole of the bladder closed in a double-layer of continuous inverting suture patterns (Cushing, 3-0 USP atraumatic poliglecaprone 25).

Retrograde filling of the bladder with sterile saline via the urinary catheter did not identify any leakage along the suture line. The abdominal cavity was thoroughly lavaged with sterile saline, the abdominal wall was closed in two layers and an abdominal bandage was applied. Hand-assisted recovery was uneventful.

### Initial postoperative care

Postoperative care included systemic antimicrobials (amikacin 25 mg/kg BW IV SID; amoxicillin 20 mg/kg BW IV TID), anti-inflammatory medication (flunixin meglumine 1.1 mg/kg BW IV BID), gastric protection (sucralfate 20 mg/kg BW PO BID) and intra-venous fluid therapy (hourly bolus infusion 500 mL Sterofundin® ISO, B. Braun, balanced electrolyte solution) with added glucose (20 mL of 40% solution, Glucose 40, B. Braun) as required.

On the day of surgery, the abdomen was found to be wet with abdominal fluid leaking from the caudal aspect of the suture line. Ultrasonographic examination confirmed the presence of free fluid within the abdomen. Sterile saline mixed with air was introduced via a urinary catheter under ultrasonographic guidance (microbubble contrast cystosonography) (27). The urinary bladder appeared intact, as both fluid and air were visible within the bladder. Abdominal fluid was evacuated and contained leucocytes 2.5 G/L, lactate 5.1 mmoL/L and creatinine 16 mg/dL. Blood work still indicated azotemia (urea 27.4 mg/dL; creatinine 3.49 mg/dL) and hyperkalemia (potassium 4.8 mmol/L), values were however improved when compared to the initial analysis. At this stage, it was suspected that residual urine might still be present in the abdominal cavity as a remnant of the previous bladder rupture. The foal was voiding urine in a physiological manner.

On the following day, the clinical and ultrasonographic picture was unchanged, and positive-contrast radiographic retrograde cystography was performed by installing contrast medium via a urinary catheter (Ultravist®, 370 mg Iod/ml, 50 mL). Laterolateral and ventrodorsal radiographic projections of the abdomen were obtained. The urinary bladder appeared intact; however, minimal accumulation of contrast medium was observed intra-abdominally between the small intestines.

**Figure 2:**
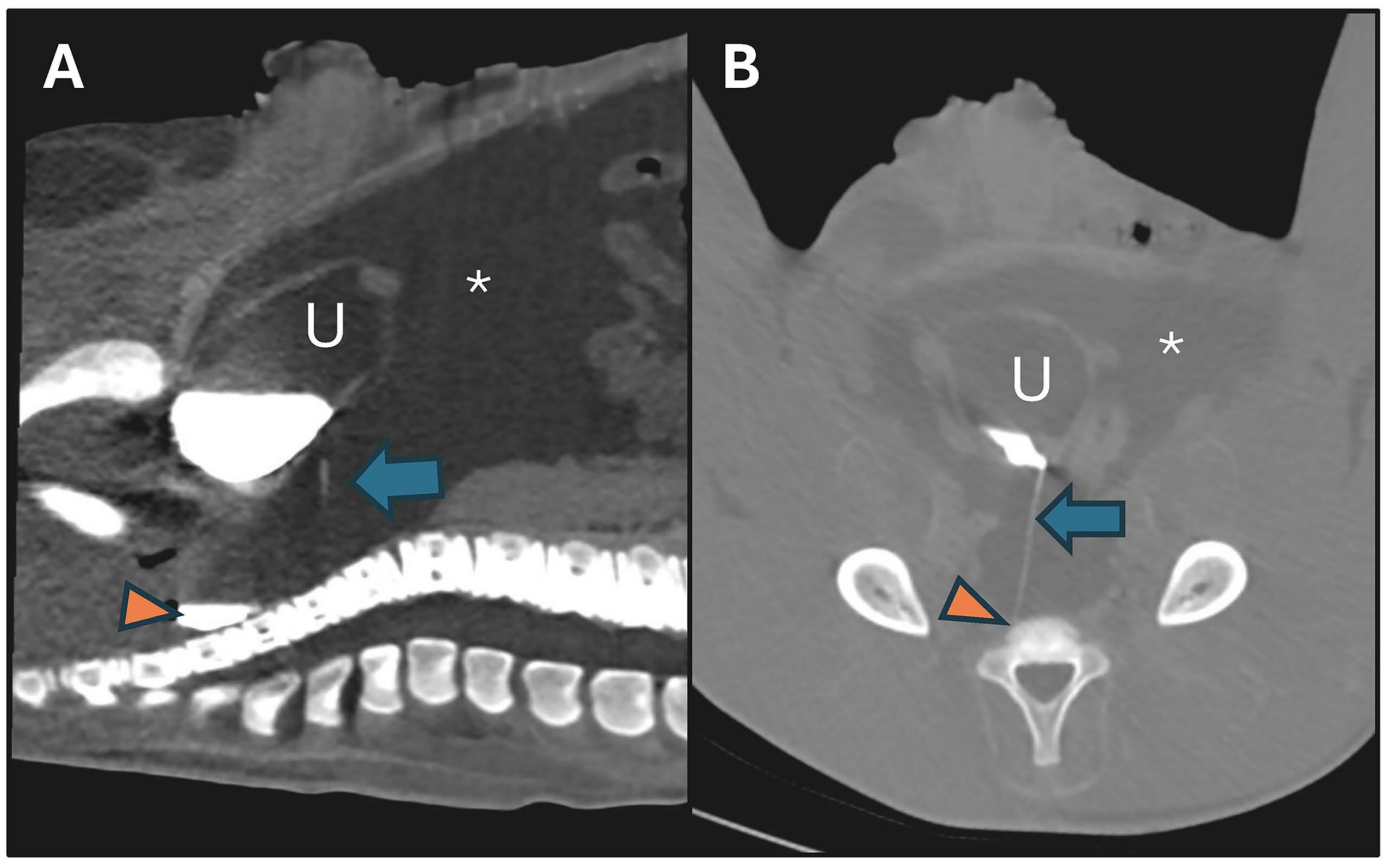
Contrast computed tomographic images (20 min after contrast administration) of the caudal abdomen of a 6-day-old Warmblood foal in dorsal recumbency following surgical repair of a dorsal wall defect of the urinary bladder. **(A)** Sagittal plane image—caudal is to the left. **(B)** Frontal plane image—ventral is to the top. Note the urinary bladder (U), dorsally filled with the hyperattenuating contrast agent and a thin, linear leakage (blue arrow) coursing toward the dorsal intraperitoneal space where contrast is pooling (orange triangle). Additionally, accumulation of free fluid is evident within the abdominal cavity (star).

Based on this finding, contrast computed tomographic (CT) examination was performed under general anesthesia two days after the initial surgery (Aquilon LB, Canon, 135kVp, 360 mAs, 1 mm slice thickness, matrix size 512 × 512, field of view 550 mm) in order to precisely localize the leakage. Following induction (see anesthetic protocol described above), a native CT scan of the caudal abdomen was obtained. For CT urography, an iodinated contrast agent (Ultravist® 370 mg iodine/ml, 120 mL, 2 mL/kg) was subsequently administered intravenously. Additional scans were acquired at 2, 7, 12, 16, 20, and 28 min after contrast administration, based on a previously described protocol, to highlight the excretory phase of the contrast medium in the urinary tract (28). CT examination demonstrated a contrast-distended urinary bladder with a thin, linear tract of extravasation extending from the dorsal vesical wall toward the intraperitoneal space. A moderate amount of free intraperitoneal fluid was further documented. There was no evidence for intra-abdominal adhesion formation (Figure 2). CT urography clearly confirmed recurrence of a defect in the urinary bladder requiring repeated surgical repair.

### Second surgery

Second surgery was preferred over continued conservative management as the foal was already anesthetized, and an insufficient repair of the bladder wall defect was very likely at this stage. The anesthetized foal was immediately transported to the surgery suite and the abdominal cavity was re-entered by suture removal. The defect in the dorsal bladder wall was found to be associated with the initial repair. Urine leakage was evident along several suture tracts where the vesical tissue was found to be inflamed and friable. The suture material was removed, and the affected wound margins were excised. Repeated repair was performed in three inverting layers (Cushing pattern, 3-0 USP atraumatic poliglecaprone 25). Additionally, the repair of the bladder apex was reinforced in simple interrupted pattern (3-0 USP atraumatic poliglecaprone 25). Retrograde distention of the bladder with methylene blue was subsequently performed to confirm the integrity of the repair. The abdominal cavity was lavaged, the ventral midline closed and dressed as described. The recovery was uneventful. An indwelling urinary catheter was maintained to prevent excessive bladder distention.

### Postoperative care after second surgery

Broad-spectrum antimicrobial therapy was continued ceftiofur (10 mg/kg BW IV BID), and the foal was administered antithrombotic medication (enoxaparin 0.35 mg/kg SC SID). The foal developed well and daily ultrasonographic assessment of the abdomen was unremarkable for three days after surgery. On day four the clinical condition of the foal deteriorated and uroperitoneum was diagnosed again. Ultrasonographic examination identified free anechoic fluid within the abdominal cavity, the urinary bladder could not be visualised and abdominocentesis yielded values consistent with uroperitoneum (peritoneal fluid urea: 34.8 mg/dL; creatinine: 7.78 mg/dL) requiring surgical intervention.

### Third surgery

**Figure 3:**
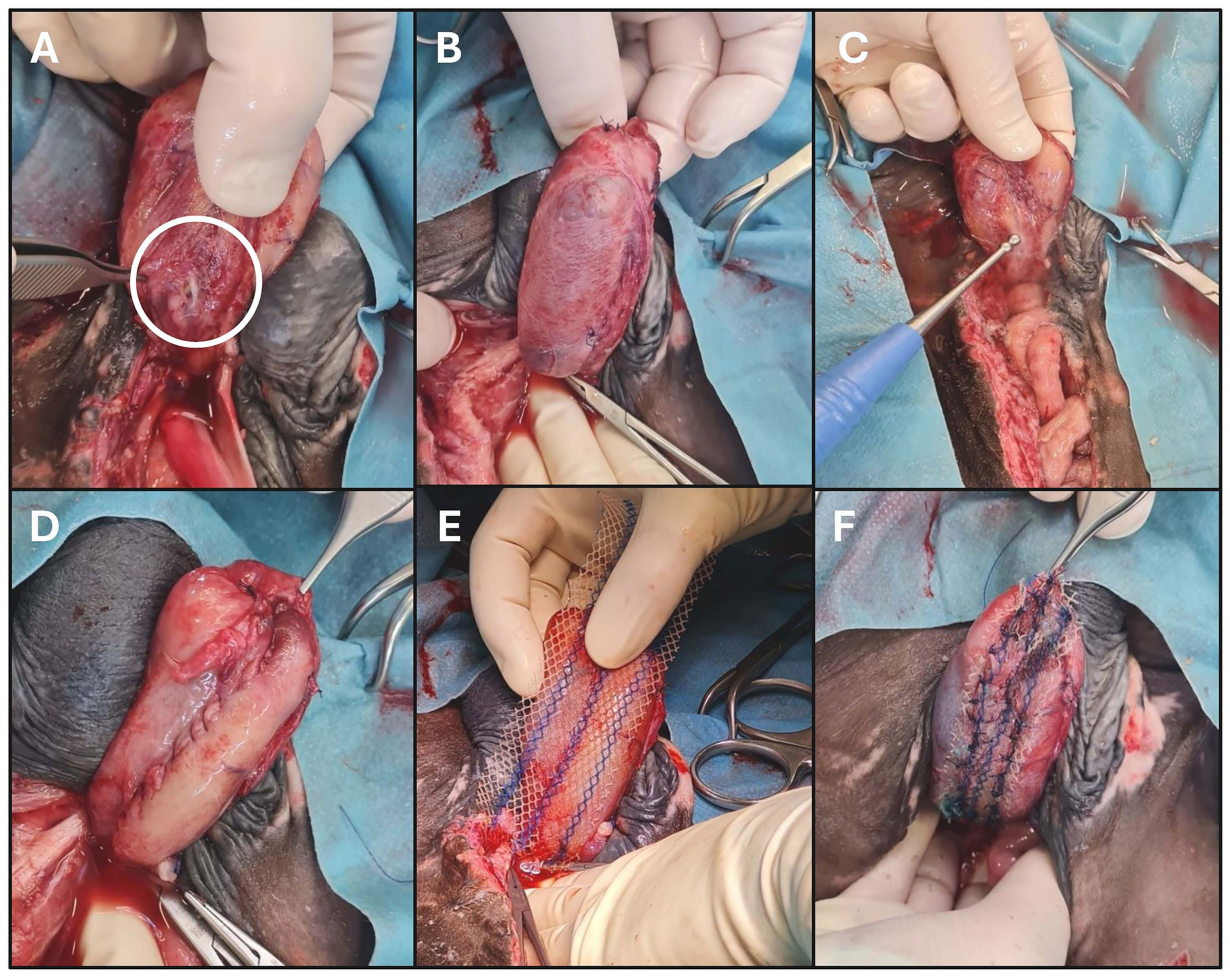
Photographic documentation of the third surgical attempt for urinary bladder wall defect closure in a male Warmblood foal. Caudal is to the top. **(A)** White circle indicates defect at the caudodorsal aspect of the bladder wall, associated with the previous repair. **(B)** Area where the dorsal vesical wall appears fragile and only the vesical mucosa is intact. **(C)** Ultrasound assisted wound debridement of the defect margins. **(D)** Defect and friable tissue in the dorsal bladder wall was sutured with Kürschner suture pattern. **(E)** Monocryl-prolene mesh placed over the suture repair. **(F)** Mesh secured with simple interrupted sutures adjacent to the suture line in the dorsal bladder wall.

Preparation including general anesthesia was performed as described. Examination of the bladder identified a small leakage associated with the previous repair (Figure 3A). All suture material was removed including the sutures at the apex of the bladder. The tissue of the dorsal bladder wall was fragile and only the vesical mucosa appeared intact (Figure 3B).

Ultrasound assisted wound debridement (Sonoca 185, Söring GmbH, Quickborn) was subsequently performed on the wound margins prior to two-layer closure of the bladder apex using Cushing suture pattern. The remaining defect, including the fragile tissue of the dorsal bladder wall, was sutured with Kürschner suture pattern (3-0 USP atraumatic poliglecaprone 25) (Figures 3C,D). A monocryl-prolene mesh (6 × 11 cm monocryl prolene composite, Ultrapro™ mesh, Ethicon) was subsequently applied over the suture line in the dorsal bladder wall (Figures 3E,F) to prevent maximal tissue expansion during distension of the bladder in this area. The mesh was secured with simple interrupted sutures (3-0 USP atraumatic poliglecaprone 25). Abdominal lavage, closure and recovery were performed routinely.

### Postoperative care after third surgery

An indwelling urinary catheter was inserted and maintained for one week postoperatively. After catheter removal the foal urinated normally. Intravenous antimicrobial and anti-inflammatory therapy were administered for four days, followed by intramuscular ceftiofur (10 mg/kg BW IM BID) and oral administration of flunixin meglumine (1.1–0.5 mg/kg BW PO BID) for an additional two weeks. Gastric protection with sucralfate (20 mg/kg BW PO BID) was maintained throughout hospitalization. As the diameter of the urinary bladder continued to appear large (7–8 cm) on repeated ultrasonographic examinations, a parasympathomimetic agent (betanechol chloride 0.4 mg/kg BW PO TID for 9 days) was added to therapy to support the bladder’s ability to contract (29). The foal was discharged in good general condition 24 days after admission (Supplementary Data Sheet 1).

Telephone follow-up confirmed that the colt had continued to void urine in normal fashion and developed physiologically with adequate weight-gain for one year after hospitalization. The animal however died peracutely at the age of 13 months. Post-mortem examination identified a 5 mm defect at the ventral aspect of the bladder. Several uroliths were however identified and were suspected to be associated with the ventral bladder wall defect. The suture repair in the dorsal bladder wall was found to be intact with the mesh integrated in the dorsal bladder wall without evidence for adhesion formation.

## Discussion

This case report describes an alternative approach for the management of recurrent uroperitoneum following surgical cystorrhaphy in a foal with bladder rupture. The use of a monocryl-prolone mesh prevented urine leakage from the bladder after two unsuccessful attempts at suture repair.

While an equine ex vivo study showed that a single layer closure using barbed suture may be sufficient to repair bladder defects, a sheep model confirmed that a double layer cystorrhaphy withstands higher pressures before leakage (30, 31). In the case described, two to three layers were used for cystorrhaphy, but uroperitoneum associated with a defect in the dorsal bladder wall still recurred.

Another study investigating complications and comorbidities in foals presenting with uroperitoneum found no impact of debridement of the bladder wound margins or the selection of suture material on recurrence or survival (3). Ultrasound assisted wound debridement was used to prepare the wound margins for repeated closure in the present case. This approach facilitates soft tissue debridement without the loss of tissue quantity. Repeated extensive debridement or excision of the wound margins potentially results in excessive tissue extension as the intravesical pressure rises. The ultrasound assisted wound debridement in combination with a two-layer closure and a monocryl-prolene mesh facilitated successful repair of recurring bladder wall defects in the described case.

The mesh used in this study was a partially absorbable mesh made of monocryl (absorbable) and prolene (non-absorbable) material. In general, mesh implantation is most frequently utilized for hernia repair in human and veterinary patients (13, 15, 32). In terms of recurrence, mesh repair is described to be superior to suture repair in human midline abdominal incisional hernias (32). Additionally, results concerning signs of pain or foreign body sensation in human patients following inguinal hernia repair suggest a low incidence of discomfort associated with the mesh repair (21). A good biocompatibility with little chronic inflammatory reaction following hernia repair with the lightweight mesh Ultrapro™ was further confirmed in a porcine model (23).

In equine surgery, implant infection is a potential complication following mesh-repair of incisional hernias, particularly as the surgical site is often infected previously and non-absorbable meshes (polypropylene mesh) are used (13, 33). Individual case reports describing mesh repair of hernias in foals including an acquired diaphragmatic and a congenital inguinal hernia are however reported with positive outcome (34, 35). One case report further describes the use of a polypropylene mesh for stabilization of a patella luxation in a foal with positive long-term outcome (16).

The intra-abdominal application as utilized for closure of the nephrosplenic space in horses is also associated with a low complication rate, where adhesion formation is the only concern (17, 18). Adhesions between the bladder and the body wall or other abdominal organs were however not detected during necropsy in the described case. Antithrombotic medication was administered following the second surgery in the described case. The risk of adhesions formation after abdominal surgery (36) as well as the thrombosis risk with prolonged intra-venous catheterisation might be reduced this way (37).

Based on the experience during management of the current case as well as the recommendation of other authors, insertion of a urinary catheter for several days should be included in the postoperative care following primary closure of a urinary bladder tear (3). Despite the risk of ascending infection, overdistension of the bladder and resulting undue stress at the site of the suture repair can potentially be prevented (26).

Extensive post-operative monitoring including repeated ultrasonographic examinations are further recommended, as the recurrence rate of vesical wall defects is high and repeated uroperitoneum is often only detected 2–4 days post-surgery (3, 4, 8, 12). It is well documented that the bladder may appear intact and intra-abdominal fluid accumulation is the only visible sign for a defect in the vesical wall during ultrasonographic examination (1, 8). Ultrasonographic visualization of bladder wall defect was only possible in 18% of cases in one study (8). The retrograde instillation of saline mixed with air in combination with ultrasonography or a radiographic contrast cystography can be performed to aid the diagnosis in inconclusive cases (9, 27). Contrast CT examination however provided the images with most diagnostic value in the case outlined here. To the best of the authors knowledge this is the first case report describing contrast CT for the diagnosis of a vesical wall defect in horses. Whilst contrast CT examination is more expensive and needs to be considered in light of the clinical condition of the foal as general anesthesia is required, it led to an accurate diagnosis and aided the decision for repeated celiotomy in the present case.

Long-term follow-up was limited by the death of the animal one year after discharge. Post-mortem examination confirmed recurrent rupture of the urinary bladder; however, unlike the previous defect, the rupture was located on the ventral aspect of the bladder wall rather than on the dorsal side. Recurrence of a bladder wall defect is the most common postoperative complication (3, 11) though this is uncommon after one year (3). Uroperitoneal recurrence may be associated with leakage along the bladder wall sutures due to bladder overpressure, latent tissue necrosis, or suture dehiscence (1, 3, 11). Chronic inflammation caused by the prolonged disease process might have contributed to an overall impaired integrity of the bladder wall (38). Intravesical suture placement has additionally been reported to promote urolith formation (3, 39). The uroliths that were identified in this case might be a sequela of the repeated attempts for suture repair. The presence of uroliths is known to cause chronic irritation with raised intravesical pressure resulting in an increasing risk for vesical rupture (40). As the mesh was attached to the serosal surface of the dorsal bladder wall and did not show any evidence for local reaction or adhesion formation it is regarded unlikely for it to have contributed to pathological tearing of the ventral aspect of the bladder.

In a case report of a calf, urinary bladder rupture was diagnosed secondary to obstruction caused by urolithiasis (41). The rupture was initially repaired by cystorrhaphy, but recurrence occurred due to necrotic tissue surrounding the tear and subsequent failure of the suture. During the second surgery, a peritoneal flap was utilized, and adjacent organs were protected from adhesions using the omentum. The calf was discharged, but follow-up examination was only available after 6 months. Foals with repeated urinary tract surgery might be at increased risk of urolith formation. Based on the outcome of the described case, follow-up examination including radiography or cystoscopy seems advisable. This way developing uroliths can be identified and removed as described elsewhere and fatal damage to the bladder wall may be prevented (3).

## Limitations

Limitations of this case report include its single-case nature, which results in a lack of comparison with alternative treatment options. Long-term outcome data were collected solely via telephone interview, which potentially limits their reliability. Additionally, as the horse died at the age of 13 months, long-term survival was inherently restricted. Due to the nature of a case report, biomechanical evaluation of the mesh on the bladder was not performed. These stated aspects would be worth investigating in a larger, systematically designed study.

## Conclusion

In conclusion, based on the experience with the described case, the challenging nature of recurrent urinary bladder wall ruptures and the difficulty of diagnosing a recurrent rupture must be carefully considered. The repeated failure of primary suture repair and the presence of friable vesical tissue can significantly increase the complexity of management. In such cases, mesh reinforcement can be a valuable salvage procedure where traditional closure techniques fail. Therefore, mesh reinforcement should be considered early in cases of recurrent bladder wall defects, or if the tissue appears fragile during the initial attempt at suture repair, to enhance structural stability and reduce the risk of recurrence. For follow-up after surgical repair of recurrent bladder rupture, the authors recommend scheduled surveillance with radiographic imaging and/or cystoscopy to assess the integrity of the repair and to identify potential uroliths at an early stage.

## Data Availability

The original contributions presented in the study are included in the article/Supplementary material, further inquiries can be directed to the corresponding author.
