# Supplementary figures and images for: Case Report: Mesh repair of a urinary bladder dorsal wall defect following repeated cystorrhaphy in a warmblood foal
Source: Front Vet Sci. 2026 Mar 31;13:1716790. doi: 10.3389/fvets.2026.1716790 (PMC13078256; doi:10.3389/fvets.2026.1716790)

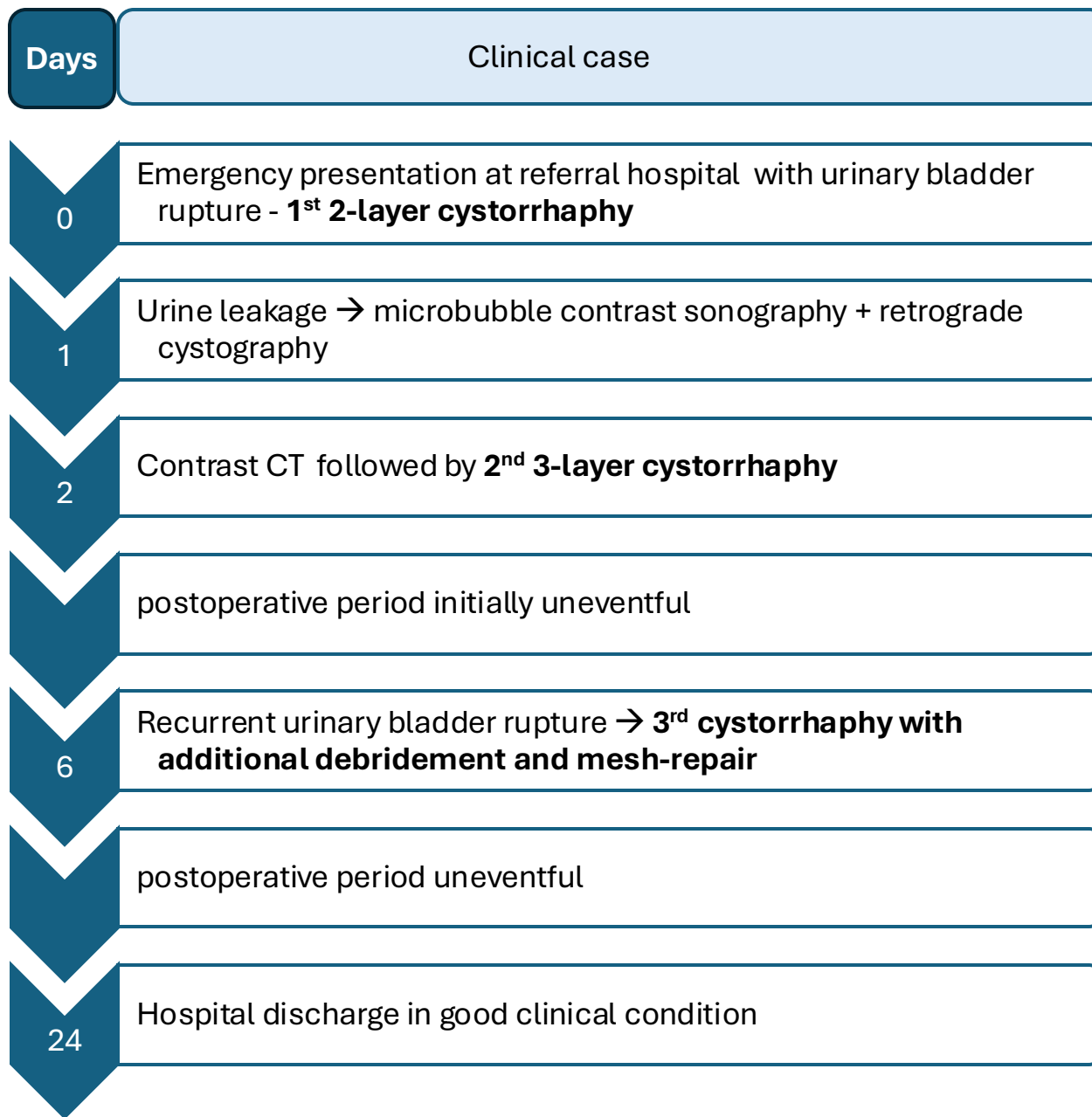

Supplement: Supplementary file 2 [file Data_Sheet_1.pdf]
